# The rOSCE: A remote clinical examination during COVID lockdown and beyond

**DOI:** 10.15694/mep.2021.000011.1

**Published:** 2021-01-14

**Authors:** Claire Stewart, Bharathy Kumaravel, Jacqueline O'Dowd, Andrew McKeown, Joanne Harris

**Affiliations:** 1 University of Buckingham

**Keywords:** Remote Digital OSCE, Clinical Competency, COVID, Undergraduate medicine, OSCE, Assessment in Medical Education

## Abstract

This article was migrated. The article was marked as recommended.

The COVID-19 pandemic has created a challenge for all medical educators. There is a clear need to train the next generation of doctors whilst ensuring that patient safety is preserved. The OSCE has long been used as the gold standard for assessing clinical competency in undergraduates ( Khan *et al*., 2013a). However, social distancing rules have meant that we have had to reconsider our traditional assessment methods.

We held a remote eight-station summative OSCE (rOSCE) for three final year resit students using Microsoft Teams. Apart from clinical examinations and practical procedures which are assessed elsewhere in our programme, the content was similar to our standard OSCE. Staff and student training ensured familiarity with the assessment modality. The rOSCE was found to be a feasible tool with high face validity.

The rOSCE is a remote assessment tool that can offer an alternative to the traditional face to face OSCEs for use in high stakes examinations. Although further research is needed, we believe that the rOSCE is scalable to larger cohorts of students and is adaptable to the needs of most undergraduate clinical competency assessments.

## Introduction

The 2020 global pandemic has forced medical educators to reflect on the viability of face to face, experiential medical education ( Sabzwari, 2013). Nevertheless, it remains imperative that we facilitate the progression of medical students, ensuring the sustainability of the future workforce that is adequately trained and assessed to preserve patient safety.

**Figure 1.**
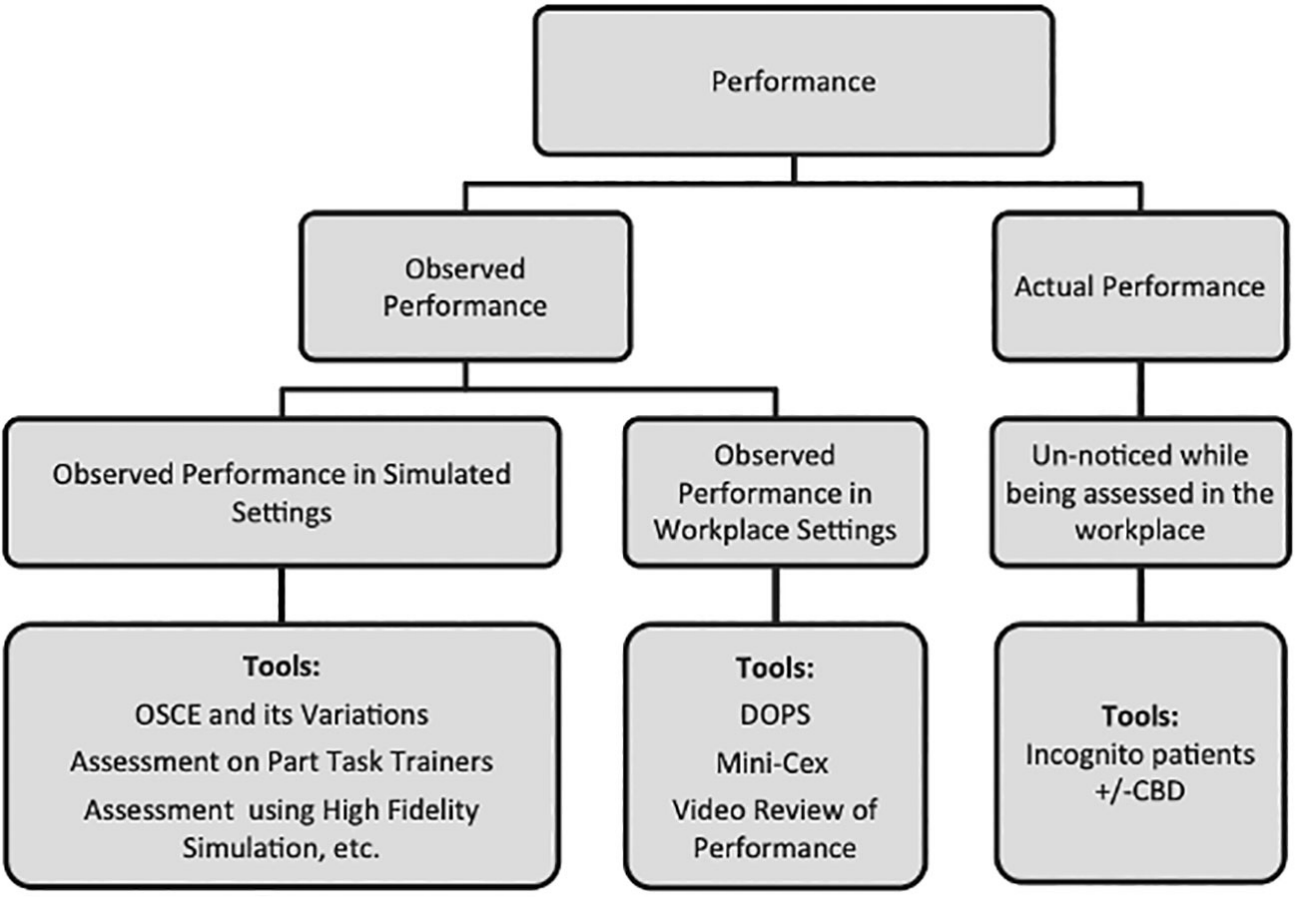
Model for the competency-based assessment of performance and examples of available assessment tools

The OSCE has been the mainstay of clinical competency assessment for years ( Khan *et al.* 2013a). Whilst its value is disputed, it remains the gold standard assessment of practical skills in medical education ( Khan, 2017). But there are limitations to the OSCE; it requires close working, detailed organisation and considerable resource in terms of clinicians, patients, physical space ( Khan *et al.,* 2013b) and costs ( Brown *et al.,* 2015). Adherence to infection control measures makes a traditional OSCE challenging during the COVID 19 pandemic. Whilst some schools may rely on Entrustable Professional Activities (EPAs) ( Cate *et al.* *,* 2015 and O’Dowd *et al.,* 2019) or workplace-based assessments ( Figure 1) to assess clinical competence, these have not been routinely applied in UK undergraduate assessment. Consequently, we could not replace the OSCE for workplace-based assessments at short notice.

( Khan and Ramachandran, 2012, reprinted by permission of Informa UK Limited, trading as Taylor & Francis Group, www.tandfonline.com)

There is limited evidence on remote summative practical assessments; one study highlighted issues about the accuracy of marks ( Novack *et al.,* 2012). Therefore, there was a need to develop a valid, feasible, remote assessment of clinical competency. We developed a new style of remote OSCE (rOSCE) using Microsoft Teams.

## The rOSCE

Although we held our finals OSCE just before the lockdown, a small number of students were required to undertake the resit rOSCE on 28 ^th^ May 2020. For this examination, following an addendum to the medical school’s Examination Regulations (Supplementary File 1), we reformatted the planned 20 stations, involving a mix of simulated and actual patients, into an 8-station rOSCE with simulated patients.

Conventional stations were modified to assess skills that could reliably be tested online. Most stations retained a simulated patient and one used a simulated doctor to allow for a handover situation. Practical procedures were removed from the rOSCE. Stations involving emergency care scenarios were modified to sequential simulations, where patient outcomes were progressively revealed through a PowerPoint presentation depending on the students’ actions. Several stations had elements of a structured interview added to overcome potential gaps in competency assessment, for instance, the patient safety aspects of prescribing. The rest of the stations remained unchanged. In order to allow for technological issues, an additional 5 minutes was allocated to each station.

Microsoft Teams (Microsoft Corporation, Redmond, Washington) was chosen as the online platform due to its versatility, familiarity and availability. A Team workspace was created with a channel for each station in the rOSCE; however, as we had fewer students than stations, we reduced the number of channels to correspond to the number of students. Each channel was managed by an invigilator and recorded; the recordings were kept within the corresponding channel, enabling moderation and external review, without disrupting the flow of the station. Despite regular use of Microsoft Teams, all stakeholders undertook a total of three hours training. A mock examination was held to familiarise the students with the process. All training, exam-day briefings and individual station calibrations were conducted via Teams.

**Figure 2.**
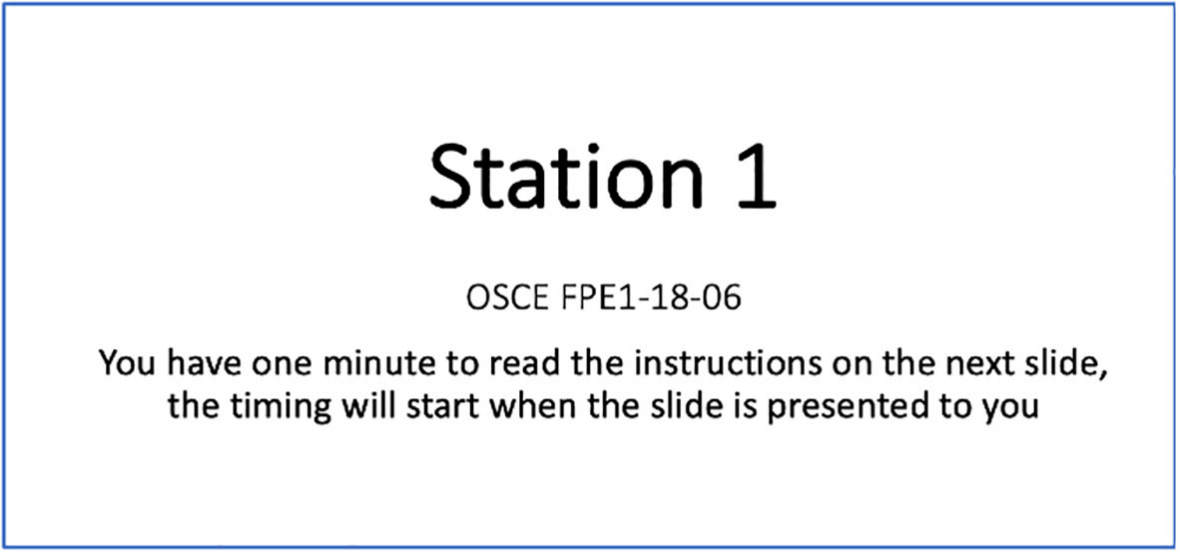
An example of a holding slide

Unlike traditional OSCEs that require students to rotate around a circuit of stations each testing different clinical competencies, the students remained in the same Teams channel throughout their entire exam. The assessors and simulated patients joined the channel intermittently during the rOSCE. When the stations began, the assessors shared a PowerPoint presentation with the students, consisting of a holding slide ( Figure 2), student instructions and any information for the station. This process removed the need for sharing of confidential material or paperwork outside the examination. The students were blocked from changing the slides using the Teams presenter functionality.

**Figure 3.**
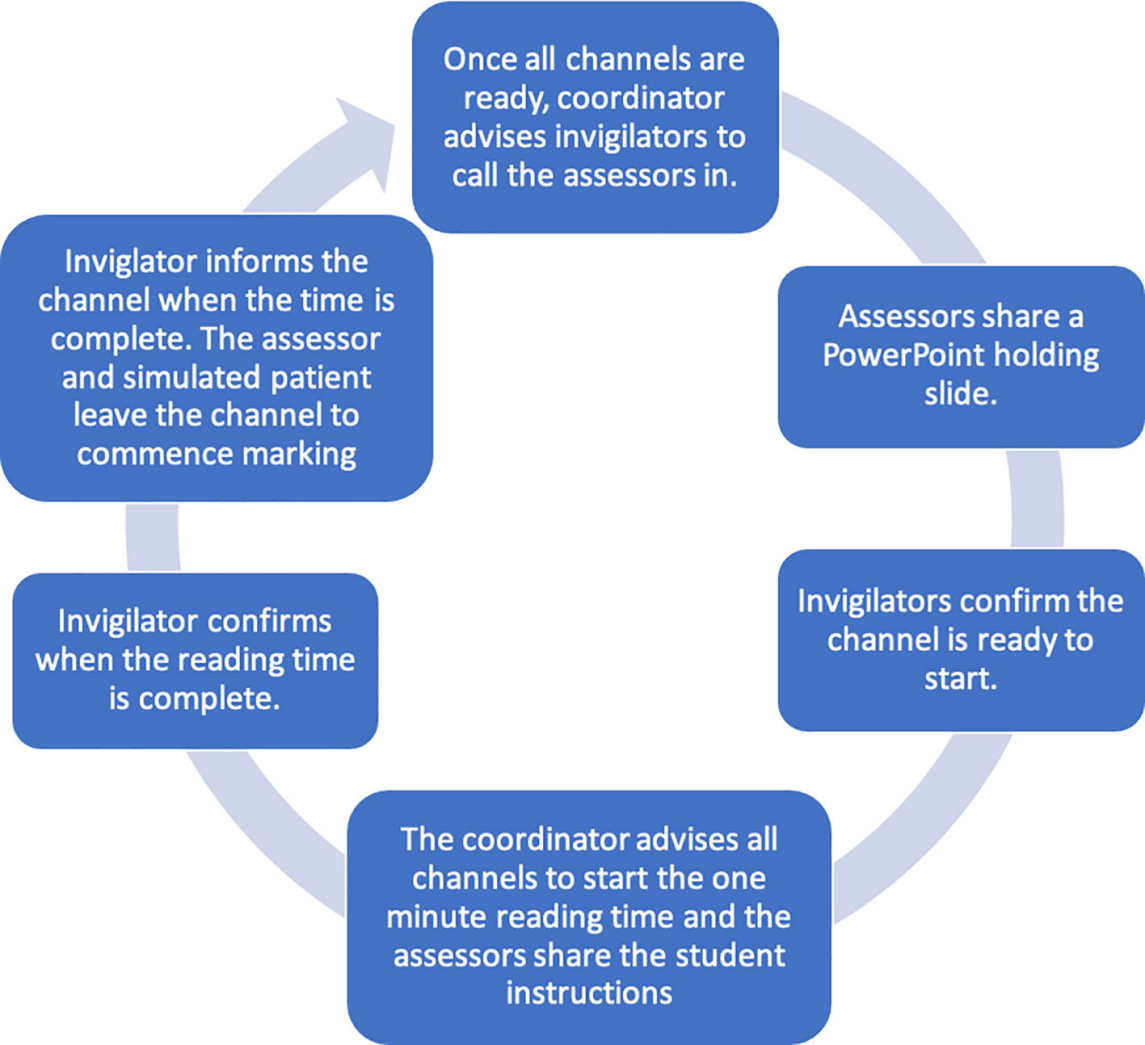
A diagram to illustrate the OSCE cycle

The simulated patients joined the channel at the start of the station, with their camera off and microphones muted until invited in. This process was repeated until the students had completed all the stations ( Figure 3).

The marking strategy did not change, we continued to use domain scoring. Marks were recorded using a Microsoft Excel file located in the MS Teams document library, however, a digital OSCE marking tool could be used if all assessors have access to the appropriate IT hardware.

Several contingencies were planned. These included an emergency contact number for the Chief Invigilator, having ‘buddy’ assessors in case of assessor illness, technology or network failure.

## Discussion

The rOSCE was completed as part of the final year resit examinations with three students and eight stations. Despite a large investment in time to plan the logistics of the assessment and training, the process was simple and easy to administer. It ran effectively and was a good test of concept. Assessors and invigilators were well prepared, and the feedback was positive. One examiner commented “ *On exam day, I was able to forget that I was in a virtual setting and concentrate on assessing the student, as in a “normal” OSCE”.* A student felt *“OSCE or rOSCE are good formats because you are also interacting with a patient”.* All examiners agreed that the rOSCE was a good strategy for assessing clinical competence.

When designing the rOSCE, we were mindful that it is unlikely that the environmental situation secondary to COVID-19 will change in the near future. Therefore, we needed to develop a system that was possible to scale up for our whole cohort rOSCEs which are planned for late 2020. Whilst further research is needed, we suspect that the rOSCE is scalable to need with an ability to run multiple circuits concurrently, removing the cost and space requirements of a traditional OSCE and the issues of standard-setting a multiple site OSCE.

Although developed for undergraduate medical students, the system could be adapted for other clinical competence assessments in both undergraduate and postgraduate settings. Removing the need for the physical presence of the student and assessor on one site also assists the development of clinical competency in remote geographical areas.

The rOSCE cannot assess practical skills or a student’s ability to elicit clinical signs but there are other reliable tools to assess these skills and therefore we have reflected on the purpose of basic practical skills stations within OSCEs. Rather than assess a student’s ability to spot clinical findings, the rOSCE should be used to assess the meta-competencies of a clinician- the ability to integrate the interpretation and analyse a situation in order the manage the patient competently.

The station design for an rOSCE requires careful planning and peer review. There is a risk that without being appropriately structured, with clear goals and outcomes, rOSCE stations could easily turn into mini vivas with all the known limitations of the viva as an assessment tool. There is also a considerable staffing requirement for the rOSCE, comparable to a conventional OSCE. It also relies on a good internet connection, though we have not found this to be a limitation despite our students being based nationally and internationally.

## Conclusion

The COVID-19 pandemic continues to challenge all facets of medical education, particularly assessment. It is vital that we continue to innovate and find ways to fill the gaps left by the lack of patient-facing assessment options during the pandemic. The rOSCE is an option to remotely assess clinical competence in undergraduate medical students providing a feasible assessment tool which is adaptable to the needs of the clinical assessment. Further research is needed to explore the potential breadth of skills examined and the scalability of this tool.

## Take Home Messages

• The challenges of the COVID-19 pandemic has caused everyone involved in medical education to think differently.
• The rOSCE was designed as a remote assessment of clinical competence run over Microsoft Teams.
• The rOSCE is a feasible tool with high face validity.
• The rOSCE can offer an alternative to the traditional face to face OSCEs for use in high stakes examinations.
• Although further research is needed, we believe that the rOSCE is scalable to larger cohorts of students and is adaptable to the needs of most undergraduate clinical competency assessments.

## Notes On Contributors

**Dr Claire Stewart** MBBS BMedSci DRCOG DFRSH FRCGP MScMedEd FAcadMEd is a senior clinical lecturer and Assessment Lead at University of Buckingham Medical School. She is a GP and has an interest in new methodologies for assessment in medical education. ORCID ID: https://orcid.org/0000-0002-7338-9809

**Dr Bharathy Kumaravel** MBBS MHSc FFPH FAcadMEd is a Senior Clinical Lecturer and Public Health Theme lead in the University of Buckingham Medical School. She is the chair of the Medical School Research Committee and the chief editor of the Journal of Medical Education Research. ORCID ID: https://orcid.org/0000-0003-4801-3081

**Dr Jacqueline O’Dowd** is the strategic development Lead at the University of Buckingham Medical School. Originally a biomedical research scientist, her current research interests lie in development and evaluation of educational interventions, change management and stakeholder engagement in evolving medical educational pedagogy and practice.

**Dr Andy McKeown** is a GP and Deputy Director of Medical Education at the University of Buckingham, focussing on the development of their Crewe Campus. His interests lie in educational authenticity, longitudinal integrated clerkships and interprofessional learning.

**Professor Joanne Harris** is Dean of the Medical School at the University of Buckingham. She is a practising GP, specialising in medical education for the past 20 years and has a particular interest in the teaching and assessment of professionalism. ORCID ID: https://orcid.org/0000-0002-1544-5585
