# Supplementary material for: The rOSCE: A remote clinical examination during COVID lockdown and beyond
Source: MedEdPublish (2016). 2021 Jan 14;10:11. [Version 1] doi: 10.15694/mep.2021.000011.1 (PMC10939528; doi:10.15694/mep.2021.000011.1)
Supplement: Peer review history

# Reviewer report: Reviewer response for version 1

**DOI:** 10.21956/mep.18840.r26847

**Published:** 2021-05-24

**Authors:** Megan Anakin

**Affiliations:** 1 University of Otago

This review has been migrated. The reviewer awarded 1 stars out of 5 Thank you for the opportunity to review your article about the use of rOSCE as an examination during lockdown. This article was of great interest to our group as it is a challenge familiar to many educators during Covid 19. The topic has merit, however more detail is required. We offer the following recommendations. To set the scene, a definition or description of a rOSCE and clinical competence is needed as these terms mean different things to different people. This definition is important as one of the findings was that all examiners agreed that the rOSCE was a good strategy for assessing clinical competence and that the system could be adapted for other clinical competence assessments in both undergraduate and postgraduate settings. The statements about Entrustable Professional Activities (EPAs) and workplace-based assessments to assess clinical competence, could be further described and related back to the appropriateness of these assessments during Covid 19. Figure 1 does not really explain these assessments and as reviewers, we were unsure as to its relevance to Covid 19. We suggest you consider deleting this figure. The comment about accuracy of marks in remote summative practical assessments sounds important and has relevance to your work (Novack et al., 2012). It would be helpful if you could provide the reader with more context about this study.Your article raised a few questions for us about how you reformatted 20 stations into an 8-station rOSCE. If practical skills were removed, please state they were assessed. Please describe the practical skills. If skills were removed explain how the skills changed the purpose of your OSCE. Including educational theory related to OSCE’s might help answer these questions and strengthen this section. Adding theory would also enhance your discussion point about reflecting on the purpose of basic practical skills stations within OSCEs and assessing the meta-competencies of a clinician. Without this context, it is difficult to determine how you reached the conclusion that “rOSCE is an option to remotely assess clinical competence in undergraduate medical students providing a feasible assessment tool which is adaptable to the needs of the clinical assessment”. We also suggest that you include citations to support your points about rOSCE stations turning into mini vivas, the known limitations of the viva as an assessment tool (what are these?), the considerable staffing requirement for the rOSCE, comparable to a conventional OSCE, and the reliance on a good internet connection. Finally, we ask you to reflect on whether you have provided the reader with enough evidence to support your take home message that the “rOSCE is a feasible tool with high face validity, that offers an alternative to the traditional face to face OSCEs for use in high stakes examinations”. We would be very happy to review a revised version of this article.

Reviewer Expertise:

NA

No decision status is available

# Reviewer report: Reviewer response for version 1

**DOI:** 10.21956/mep.18840.r26849

**Published:** 2021-01-24

**Authors:** Mohamed Al-Eraky

**Affiliations:** 1 University of Dammam

This review has been migrated. The reviewer awarded 3 stars out of 5 Thank you for sharing this interesting study. I agree with Julie that authors may need to explain the rationale behind decreasing the number of stations from 20 to only 8. They may also share the approach they’ve used to shortlist these eight skills. I also think there will be no major problem in assessing communication skills using simulated patients (as for history taking) or simulated doctors (as for handover). The big challenge in assessment of practical procedures, which could not be addressed. Yet, the current study may serve as the basis to develop ‘virtual patients’ to assess clinical reasoning and decision-making skills. I encourage authors to use their expertise to plan rOSCE to assess practical procedures, which is an urgent need of the hour in medical education. Good luck!

Reviewer Expertise:

NA

No decision status is available

# Reviewer report: Reviewer response for version 1

**DOI:** 10.21956/mep.18840.r26848

**Published:** 2021-01-14

**Authors:** Julie Hunt

**Affiliations:** 1 Lincoln Memorial University

This review has been migrated. The reviewer awarded 4 stars out of 5 This article describing a remote OSCE will be of interest of those who are teaching and assessing clinical skills during the pandemic. The authors do a good job of describing how they used Microsoft Teams to run a resit OSCE, though only having run 3 students through the exam, this can only be considered pilot data. I would like to have seen the authors discuss the decision to go from the standard 20-station OSCE to the 8-station rOSCE, as decreasing the number of stations reduces the reliability of the exam.For transparency, I am an Associate Editor of MedEdPublish. However I have posted this review as a member of the review panel with relevant expertise and so this review represents a personal, not institutional, opinion.

Reviewer Expertise:

NA

No decision status is available
